# Leveraging CD4 Cell Count at Entry Into Care to Monitor Success of Human Immunodeficiency Virus Prevention, Treatment, and Public Health Programming in the Greater St Louis Area Between 2017 and 2020

**DOI:** 10.1093/ofid/ofad477

**Published:** 2023-09-18

**Authors:** Lindsey M Filiatreau, Aaloke Mody, Daniel Vo, Cory Bradley, Aditi Ramakrishnan, Julia López, Jane O’Halloran, Anne Trolard, William G Powderly, Elvin H Geng

**Affiliations:** Division of Infectious Diseases, School of Medicine, Washington University, St Louis, Missouri, USA; Division of Infectious Diseases, School of Medicine, Washington University, St Louis, Missouri, USA; Division of Infectious Diseases, School of Medicine, Washington University, St Louis, Missouri, USA; Division of Infectious Diseases, School of Medicine, Washington University, St Louis, Missouri, USA; Division of Infectious Diseases, School of Medicine, Washington University, St Louis, Missouri, USA; Division of Infectious Diseases, School of Medicine, Washington University, St Louis, Missouri, USA; Division of Infectious Diseases, School of Medicine, Washington University, St Louis, Missouri, USA; Division of Infectious Diseases, School of Medicine, Washington University, St Louis, Missouri, USA; Division of Infectious Diseases, School of Medicine, Washington University, St Louis, Missouri, USA; Division of Infectious Diseases, School of Medicine, Washington University, St Louis, Missouri, USA

**Keywords:** CD4 cell count, EHE, HIV, ending the HIV epidemic, linkage to care

## Abstract

CD4 cell count at entry into human immunodeficiency virus (HIV) care is a useful indicator of success of multiple steps in HIV public health programming. We demonstrate that CD4 cell count at care initiation was stable in St Louis between 2017 and 2019 but declined in 2020. Missouri efforts in the Ending the HIV Epidemic plan should focus on rapidly identifying individuals with undiagnosed HIV infection.

In persons living with human immunodeficiency virus (HIV), CD4 cell count at entry into care is an important and underused indicator of success of multiple steps in HIV public health programming, including the reach of HIV testing programs and the completeness and timeliness of linkage to HIV care [1]. Ultimately, higher CD4 cell counts at entry into care indicate reduced time between seroconversion and initiation of antiretroviral treatment (ART). From a clinical perspective, reduced time between seroconversion and ART initiation reduces the risk of adverse health outcomes (eg, renal impairment and cardiac events) even when CD4 cell counts are above the range where frank immunodeficiencies are present [2]. From a public health perspective, this also reduces “community viral load,” which minimizes susceptibility of new infections [3].

Missouri is among the 7 states identified as high priority in the Ending the HIV Epidemic (EHE) plan owing to a substantial number of HIV diagnoses in 2017 and 2018 [4]. As additional efforts are made to address barriers to improved HIV care outcomes across the state, robust monitoring of appropriate metrics is necessary to understand the success of these efforts. Currently, reporting by Missouri public health authorities describes the numbers of persons known to be living with HIV and with new HIV diagnoses. However, these figures do not provide information on how effectively or efficiently persons with HIV are receiving diagnoses or being linked to care following infection—a gap directly addressed through monitoring CD4 cell counts at care or ART initiation.

In the past 2 decades, CD4 cell counts at treatment initiation have risen globally, suggesting improved public health programming that has minimized the time between seroconversion and ART initiation [5, 6]. Understanding the CD4 trend at care entry in St Louis and how it compares regionally and globally will elucidate the success or failure of EHE efforts to reduce late diagnoses and the potential impact of coronavirus disease 2019 (COVID-19), which disrupted healthcare globally. Moreover, identifying sociodemographic correlates of CD4 at care entry can aid in identifying those who might benefit most from targeted testing and linkage efforts.

## METHODS

### Study Population and Setting

We leveraged electronic health record data from patients at the Washington University Infectious Diseases Clinic in St Louis, Missouri. Missouri is a largely rural Midwestern state of approximately 6 million residents, 82% of whom are white [7]. Nearly 14 000 Missourians are known to be living with HIV, and approximately 45% of these individuals live in the St Louis region [8].

The Washington University Infectious Diseases clinic is the largest provider of HIV primary care and Ryan White Part C grantee in the region and offers a host of integrated healthcare services (eg, case management and laboratory testing) [9]. We extracted electronic health record data for all individuals seeking care between January 2017 and December 2020 (n = 3131). Individuals who were new or presumed new to care (n = 395) were included. Individuals without a documented CD4 cell count or viral load measurement within 90 days of their recorded diagnosis date and those with a viral load <1000 copies/mL at entry were excluded, as they were hypothesized to have been on ART previously (n = 145).

### Participant Consent

This was a secondary data analysis that leveraged existing electronic health records. As such, no patient consent was obtained. Secondary data analysis was approved by the Washington University Institutional Review Board.

### Measures

We defined CD4 cell count at entry into care as the first documented CD4 cell count measurement taken within 90 days of diagnosis. CD4 cell count was considered as a continuous measure and dichotomized to represent those with late-stage entry into care (CD4 cell count <200/μL) versus others (CD4 cell count ≥200/μL). Demographic and clinical characteristics of interest included age, sex, race/ethnicity, HIV susceptibility category, and year of diagnosis.

### Statistical Analyses

Counts and proportions or medians and interquartile ranges (IQR) were used to describe the demographic and clinical characteristics of the study population overall and by year of diagnosis. Wilcoxon rank sum tests were used to compare median CD4 cell count at entry into care by year. Separate binomial regression models were used to estimate prevalence differences and ratios to assess absolute and relative discrepancies in late-stage entry into care by year of diagnosis and patient demographic and clinical characteristics. All analyses were conducted using SAS v.9.4 software (SAS Institute).

## RESULTS

Of the 250 included individuals who entered care in the Washington University Infectious Disease Clinic between 2017 and 2020, a majority were male (n = 183 [73.2%]), black, non-Hispanic (n = 182 [72.8%]), and men who have sex with men (n = 148 [59.2%]). Nearly one-third were aged 18–24 years at entry (n = 77 [30.8%]). Sixty individuals (24.0%) had HIV infection diagnosed in 2017, 58 (23.2%) in 2018, 73 (29.2%) in 2019, and 59 (23.6%) in 2020. The median CD4 cell count at entry into care across the study period was 361/μL (IQR, 230–550/μL). Just over 20% (n = 55 [22.0%]) had a CD4 cell count <200/μL at entry.

**Figure 1.**
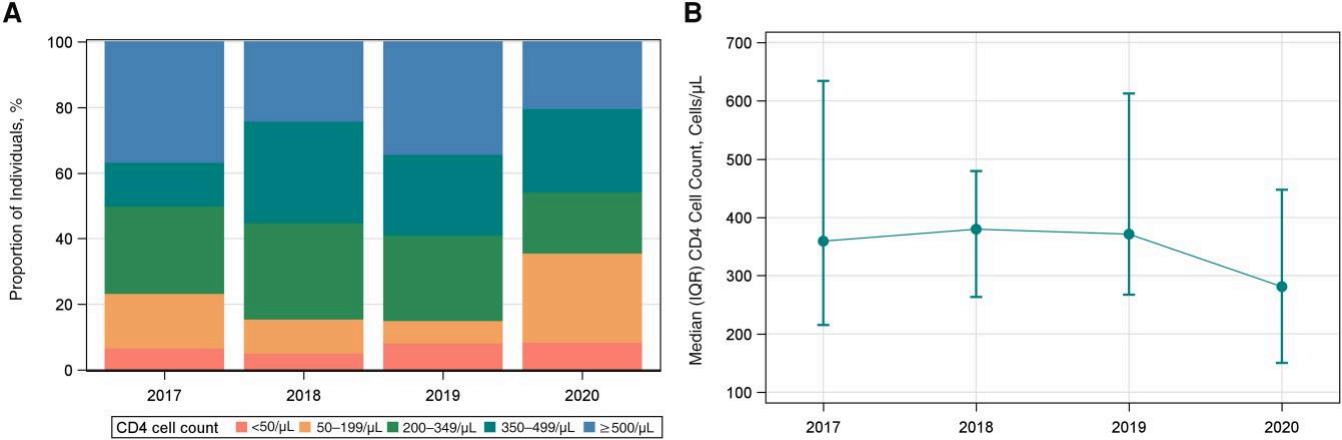
Distribution of CD4 cell count (*A*) and median CD4 cell count and interquartile range (IQR) (*B*) among individuals with newly diagnosed human immunodeficiency virus (HIV) infection initiating care in the Washington University Infectious Diseases Clinic between 2017 and 2020.

The median CD4 cell count (IQR) was 359/μL (214–636/μL) in 2017, 380/μL (262–481/μL) in 2018, 371/μL (266–614/μL) in 2019, and 281/μL (149–449/μL) in 2020. The median CD4 cell count at entry into care was lower in 2020 compared with either 2017 (*z* = −1.93; *P* = .05) or 2018 (*z* = −2.49; *P* = .01). The proportion of individuals with a CD4 cell count <200/μL was 23.3% (n = 14) in 2017, 15.5% (n = 9) in 2018, 15.1% (n = 11) in 2019, and 35.6% (n = 21) in 2020 (Figure 1).

Age was associated with late-stage entry into care in regression analyses (Table 1). For every 5-year increase in age, the relative prevalence of late-stage entry into care increased by a magnitude of 1.12 (95% confidence interval, 1.04–1.20). The absolute prevalence of late-stage entry into care increased by a magnitude of 3 percentage points (risk difference, 0.03 [95% confidence interval, .01–.06). No meaningful association was observed between sex, race/ethnicity, or HIV susceptibility category and late-stage entry into care.

**Table 1.** Estimates of Relative and Absolute Associations Between Clinical and Sociodemographic Characteristics and Late-Stage Entry to Care Among 250 Persons Entering Human Immunodeficiency Virus Care in the Greater St Louis Area, 2017–2020

Characteristic	Prevalence Ratio (95% CI)^a^	Prevalence Difference (95% CI)^a^
Sex		
Male	1.00 (Ref)	0.00 (Ref)
Female	0.94 (.54–1.64)	−0.01 (−.13 to .11)
Transgender^b^	…	…
Age^c^	1.12 (1.04–1.20)	0.03 (.01–.06)
Race/ethnicity		
White, non-Hispanic	1.00 (Ref)	0.00 (Ref)
Black, non-Hispanic	1.25 (.68–2.32)	0.05 (−.07 to .17)
Other	0.71 (.17–2.88)	−0.06 (−.26 to .15)
Year of entry into care		
2017	1.00 (Ref)	0.00 (Ref)
2018	0.67 (.31–1.42)	−0.08 (−.22 to .06)
2019	0.65 (.32–1.32)	−0.08 (−.22 to .05)
2020	1.53 (.86–2.71)	0.12 (−.03 to .29)
HIV susceptibility		
Heterosexual	1.00 (Ref)	0.00 (Ref)
MSM	0.97 (.60–1.56)	−0.01 (−.11 to .10)
Other	0.39 (.05–2.65)	−0.14 (−.33 to .05)

Abbreviations: CI, confidence interval; HIV, human immunodeficiency virus; MSM, men who have sex with men; Ref, reference.

a Modeling prevalence of late-stage entry into care (CD4 cell count, <200/μL).

b No transgender individuals entered care with a CD4 cell count <200/μL.

c Estimate for 5-year increase in age.

## DISCUSSION

Timely diagnosis and entry into care are critical to ending the HIV epidemic in the United States. While the EHE initiative holds promise for reinvigorating efforts to eliminate HIV nationally, use of inappropriate monitoring and evaluation metrics may provide a skewed or misguided understanding of the true performance of EHE strategies over time. We harnessed data from the largest HIV care provider in the greater St Louis area to demonstrate the utility of CD4 cell count at entry into care as a measure of the success of public health programming for HIV and in identifying subgroups most at risk for delayed diagnosis and linkage to care.

Data suggest that, across the study period, the COVID-19 pandemic likely hindered progress in improving the timeliness of HIV diagnosis and entry into care. Between 2017 and 2020, the lowest median CD4 cell count at entry (281/μL) and highest proportion of individuals with a CD4 cell count <200/μL at entry (35.6%) was observed among those with HIV diagnosed in 2020, despite no meaningful differences in the absolute number of diagnoses across years. Age was the only factor explored that was meaningfully associated with late-stage entry into care, with older individuals more likely to enter later.

It has been posited that, nationally, we are closest to achieving the EHE diagnosis goals (ie, diagnose all individuals with HIV as early as possible following infection) [10]. While data from America's HIV Epidemic Analysis Dashboard suggest that the number of new HIV diagnoses in Missouri declined between 2017 and 2020 [11], we observed no meaningful differences in the annual number of diagnoses across this period, including in 2020 when diagnoses declined nationally owing to foregone care seeking attributable to COVID-19 [12]. Our findings also demonstrate that individuals initiating care in the Washington University Infectious Diseases Clinic in 2020 presented for care *later* than those who initiated in either 2017 or 2018. Despite consistency in the number of new diagnoses across years, these differences could represent later presentations due to COVID-19–related care disruptions (ie, delays in testing or linkage to care).

While the median CD4 cell counts at care initiation observed in our study are similar to nationally reported estimates [6], substantial disparities are noted when comparing our results with those in more metropolitan areas with sustained HIV prevention resources and robust monitoring systems [13, 14]. For example, the median CD4 cell count at entry into care in 2020 was 439/μL in San Francisco [14] and 374/μL in King County, Washington [13], while our estimate was 281/μL. This suggests that, in addition to improving resource availability for HIV prevention, identification of individuals with undiagnosed HIV infection and more robust linkage to care programs may need greater prioritization in the St Louis area to minimize community viral load and new infections and achieve equitable outcomes relative to other metropolitan areas. Additional data from 2021 and beyond are also needed to ascertain whether these trends have continued as routine healthcare seeking resumed.

Finally, as has been observed in other settings, older age was associated with late-stage entry into HIV care, suggesting that older adults who have never tested or have engaged in HIV-susceptible behaviors since their last test may be particularly important to target in outreach programs [15, 16]. A host of strategies have been identified to improve HIV testing uptake across the United States and should be urgently used to improve timing of HIV diagnosis in Missouri, particularly among older populations [10].

In summary, the timing of entry into HIV care in St Louis has not improved since the launch of the EHE initiative, with the poorest outcomes observed during the COVID-19 pandemic. If we are to end the HIV epidemic in the United States by 2030, robust metrics must be harnessed to monitor the effectiveness of EHE strategies in priority jurisdictions such as Missouri, as well as nationally.

## References

[1] Nash D , Robertson M. How to evolve the response to the global HIV epidemic with new metrics and targets based on pre-treatment CD4 counts. Curr HIV/AIDS Rep 2019; 16:304–13. 31278620 10.1007/s11904-019-00452-7 PMC10938289

[2] El-Sadr WM , Lundgren JD, Neaton JD, et al; Strategies for Management of Antiretroviral Therapy (SMART) Study Group; CD4+ count-guided interruption of antiretroviral treatment. N Engl J Med 2006; 355:2283–96. 17135583 10.1056/NEJMoa062360

[3] Kranzer K , Lawn SD, Johnson LF, Bekker LG, Wood R. Community viral load and CD4 count distribution among people living with HIV in a South African township: implications for treatment as prevention. J Acquir Immune Defic Syndr 2013; 63:498–505. 23572010 10.1097/QAI.0b013e318293ae48 PMC4233323

[4] Fauci AS , Redfield RR, Sigounas G, Weahkee MD, Giroir BP. Ending the HIV epidemic: a plan for the United States. JAMA 2019; 321:844–5. 30730529 10.1001/jama.2019.1343

[5] The IeDEA and COHERE Cohort Collaborations . Global trends in CD4 cell count at the start of antiretroviral therapy: collaborative study of treatment programs. Clin Infect Dis 2018; 66:893–903. 29373672 10.1093/cid/cix915 PMC5848308

[6] Lee JS , Humes EA, Hogan BC, et al CD4 count at entry into care and at antiretroviral therapy prescription among adults with human immunodeficiency virus in the United States, 2005–2018. Clin Infect Dis 2021; 73:e2334–7. 33383586 10.1093/cid/ciaa1904 PMC8492212

[7] Kaur R , Depue S. Missouri state epidemiological profile 2021. Missouri Institute of Mental Health, 2021. Available at: http://archive.org/details/2021MOEpiProfile. Accessed 10 August 2023.

[8] Hellman L . Fast Track Cities HIV Data. Missouri Department of Health & Senior Services, 2023. Available at: https://www.stlouis-mo.gov/hiv/data/totals.cfm. Accessed 1 May 2023.

[9] Division of Infectious Diseases HIV Program. Available at: https://hiv.wustl.edu/about-us/. Accessed 5 May 2022.

[10] Delaney KP , DiNenno EA. HIV testing strategies for health departments to End the epidemic in the U.S. Am J Prevent Med 2021; 61:S6–S15. 10.1016/j.amepre.2021.06.002 PMC9552039 34686292

[11] Missouri. Available at: https://ahead.hiv.gov/locations/missouri. Accessed 5 May 2022.

[12] DiNenno EA . HIV testing before and during the COVID-19 pandemic—United States, 2019–2020. MMWR Morb Mortal Wkly Rep 2022; 71:820–4. 35737573 10.15585/mmwr.mm7125a2

[13] Washington State and King County . HIV/AIDS epidemiology report and community profile. 2021. Available at: https://kingcounty.gov/~/media/depts/health/communicable-diseases/documents/hivstd/2021-hiv-aids-epidemiology-annualreport.ashx?la=en. Accessed 10 December 2022.

[14] San Francisco Department of Public Health Population Health Division . HIV epidemiology annual report 2020. 2021. Available at: https://www.sfdph.org/dph/files/reports/RptsHIVAIDS/AnnualReport2020-Purple_20210817Web.pdf. Accessed 5 May 2022.

[15] Buchacz K , Armon C, Palella FJ, et al CD4 cell counts at HIV diagnosis among HIV outpatient study participants, 2000–2009. AIDS Res Treat 2011; 2012:e869841. 10.1155/2012/869841 PMC3176626 21941640

[16] Means AR , Risher KA, Ujeneza EL, Maposa I, Nondi J, Bellan SE. Impact of age and sex on CD4+ cell count trajectories following treatment initiation: an analysis of the Tanzanian HIV treatment database. PLoS One 2016; 11:e0164148. 10.1371/journal.pone.0164148 PMC5055355 27716818

